# A Novel ceRNA Regulatory Network Involving the Long Non-Coding Antisense RNA SPACA6P-AS, miR-125a and its mRNA Targets in Hepatocarcinoma Cells

**DOI:** 10.3390/ijms21145068

**Published:** 2020-07-17

**Authors:** Armando Di Palo, Chiara Siniscalchi, Nicola Mosca, Aniello Russo, Nicoletta Potenza

**Affiliations:** 1 Department of Environmental, Biological and Pharmaceutical Sciences and Technologies, University of Campania “Luigi Vanvitelli”, 81100 Caserta, Italy; armando.dipalo@unicampania.it (A.D.P.); chiara.siniscalchi@unicampania.it (C.S.); aniello.russo@unicampania.it (A.R.); 2 Inserm, BMGIC, U1035, University of Bordeaux, 33076 Bordeaux, France; nicola.mosca@u-bordeaux.fr

**Keywords:** miR-125a, SPACA6P-AS, non-coding RNA, ceRNET, cancer

## Abstract

MicroRNAs (miRNA), and more recently long non-coding RNAs (lncRNA), are emerging as a driving force for hepatocellular carcinoma (HCC), one of the leading causes of cancer-related death. In this work, we investigated a possible RNA regulatory network involving two oncosuppressive miRNAs, miR-125a and let-7e, and a long non-coding antisense RNA, SPACA6P-AS (SP-AS), all transcribed from the same locus, with SP-AS in the opposite direction and thus carrying complementary sequences to the miRNAs. In vitro experiments validated the binding of the miRNAs to SP-AS. Then, the boosting of either the miRNAs or SP-AS levels demonstrated their reciprocal inhibition. In addition, overexpression of SP-AS resulted in a reduced silencing activity of miR-125a and let-7e toward their key oncogenic targets, i.e., Lin28b, MMP11, SIRT7, Zbtb7a, Cyclin D1, CDC25B, HMGA2, that resulted significantly upregulated. Finally, the analysis of 374 HCC samples in comparison to 50 normal liver tissues showed an upregulation of SP-AS and a reverse expression of miR-125a, not observed for let-7e; consistently, miR-125a oncogenic targets were upregulated. Overall, the data depict a novel competing endogenous RNA (ceRNA) network, ceRNET, whereby miR-125a can regulate the expression of SP-AS, which in turn regulates the miRNA by competing with the binding to the mRNA targets. We speculate that the unbalancing of any network component may contribute to hepatocarcinogenesis.

## 1. Introduction

Hepatocellular carcinoma (HCC) is one of the most common aggressive human malignancies, ranking third for cancer-related death and fifth most common solid tumor worldwide [1]. More than 80% of human HCCs arise on a consequence of chronic liver disease resulting from viral hepatitis, metabolic syndrome, alcohol abuse, exposure to carcinogenic agents, and genetic disease such as Wilson’s disease and hemochromatosis [2]. Different molecular mechanisms responsible for initiating and promoting HCC have been identified, such as receptor tyrosine kinase pathways, the Ras mitogen-activated protein kinase (Ras/Raf/MAPK), the phosphatidylinositol 3-kinase (PI3K)/AKT/mammalian target of rapamycin (mTOR), the Wnt/β-catenin signaling pathway, the ubiquitin/proteasome degradation, and the hedgehog signaling pathway [3].

Besides the well-known role of oncogenic or tumor suppressor proteins, functional studies have also demonstrated the active involvement of non-coding RNA (ncRNA), mainly microRNAs (miRNA) and more recently long non-coding RNAs (lncRNA), in the regulation of key pathways driving hepatocarcinogenesis, through the direct and indirect targeting of crucial oncogenes or tumor-suppressor genes [4]. In particular, miRNAs have been involved at the different levels of hepatocarcinogenesis, from early development to advanced stages, with some having an oncogenic role and others a tumor suppressor activity [5,6,7]. Moreover, miRNAs signatures (both tissue and circulating) have been associated with increased HCC risk, neoplastic development, advanced stages, and vascular invasion, and some of them were confirmed in rodent models and tested as therapeutic targets [8,9,10].

With regard to lncRNAs, they are emerging as novel players in HCC [11,12]. They are non-coding RNAs more than 200 nt long with limited protein coding potential, generally classified on the basis of their genomic location or function [13,14,15]. Like miRNAs, they can also be secreted and found in the extracellular space [16]. Physiologically, lncRNAs participate in gene expression modulation at multiple levels, either in the nucleus, by interacting with DNA, chromatin modifying complexes, and transcriptional regulators, and in cytoplasm, acting as sponges for other RNA molecules, proteins, and regulating mRNA degradation and translation [17]. In particular, they can also act as a sponge or competing endogenous RNAs (ceRNA) for miRNAs, thus protecting target mRNAs from the miRNA interfering activity, and giving rise to complex regulatory RNA-based circuits, with an impact on different physiological and pathological processes [18]. In those ceRNA networks (ceRNET), comprising lncRNAs, miRNAs, and mRNAs, miRNAs and lncRNAs are likely to modulate each other, since miRNAs can regulate the expression of lncRNAs, which in turn can regulate miRNAs competing with the binding to mRNA targets [19,20]. The unbalancing of any network component could result in pathological consequences.

Regarding miRNAs and HCC, different independent studies focused the attention on miR-125a, endowed with an oncosuppressive role in different tumors, including HCC [21,22]. In fact, miR-125a has an antiproliferative activity towards HCC cells, is able to mediate the activity of Sorafenib, a drug representing the standard of care for advanced HCC, is downregulated in HCC biopsies in comparison to adjacent non-tumoral tissues and is able to limit tumor growth, in vitro and in vivo, by downregulating different targets such as matrix metalloproteinase-11 (MMP11), sirtuin-7 (SIRT-7), Zbtb7a, and also the stemness factor Lin28b [23,24,25,26,27,28,29,30,31].

**Figure 1:**
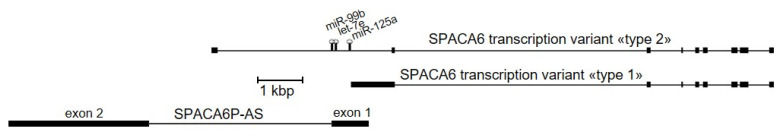
Human Spaca6/microRNA (miRNA) cluster locus. Top lines show the two main transcripts of SPACA6 gene, each yielding different splice variants. The bottom line refers to SP-AS that is transcribed in the opposite direction. Exons and miRNAs are indicated by black boxes and loops, respectively.

miR-125a genomic sequence belongs to the chromosome 19 and is clustered with two other microRNAs, miR-99b and let-7e (Figure 1) [32]. Both miRNAs are also reported to have tumor suppressor activity; in particular, let-7e belongs to the large let-7 family of oncosuppressive miRNAs acting on key oncogenes, such as Ras and Myc [33], but also HMGA2, CDC25B in HCC [34], and indeed Lin28 [35]. The miRNA cluster is placed in the first intron of a gene producing different spliced variants encoding SPACA6 (sperm acrosome associated 6) protein isoforms, involved in the egg-sperm fusion during fertilization in mice [36,37,38]. Recently, a long non-coding RNA transcribed in the antisense direction of SPACA6, thus named SPACA6P-AS (SP-AS), has been reported (NR_108100) and validated only from human locus [39]. It consists of two exons, the first 823 nt-and the second 3139 nt-long; the intron is 4134 nt long. The first exon comprises fully complementary sequences to miR-125a and let-7e; miR-99b complementary sequence resides at the 5′ splice junction of the intron of SP-AS (Figure 1).

Based on genomic location and full complementary of the miRNAs to mature SP-AS, as well as the tumor suppressor activity of the miRNAs, in this work we investigated the hypothesis that SP-AS, miR-125a, let-7e and their target transcripts depict a novel ceRNET, with a possible role in hepatocarcinogenesis.

## 2. Results

### 2.1. Expression of miR-125a, let-7e, and SP-AS in Liver Cells

**Figure 2:**
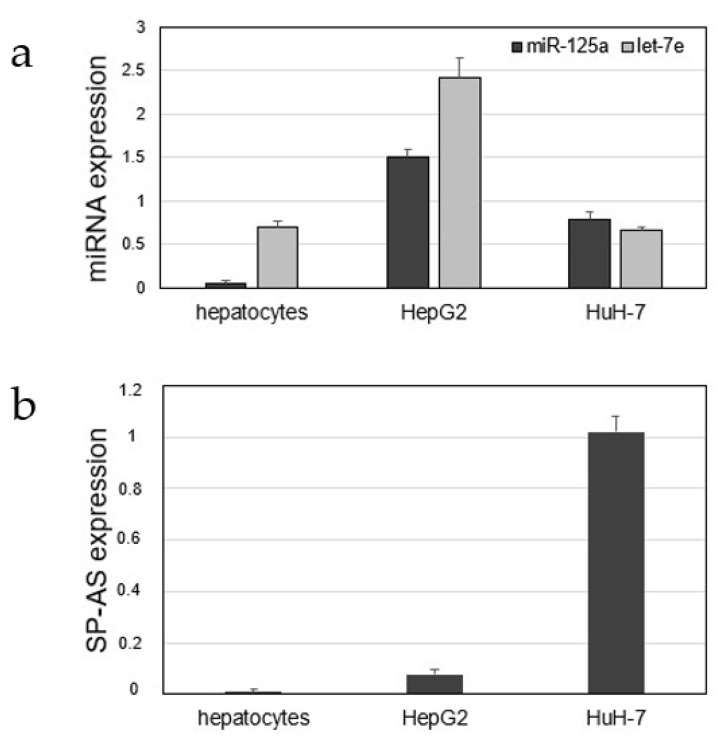
Expression of miR-125a, let-7e, and SP-AS in primary hepatocytes and hepatocellular carcinoma (HCC) cell lines HepG2 and HuH-7. Expression of the miRNAs (**a**) and SP-AS (**b**) was determined by TaqMan or SYBR Green qPCR assays, respectively; data from triplicate analyses were reported as mean ± SD of 2^Δ^^Ct^ values. Values for SP-AS expression are given multiplied by 10^4^.

Before proceeding to validate the possible functional interaction between the miRNAs and the lncRNA SP-AS, we determined their expression in normal human primary hepatocytes and in the commonly used HCC cell lines, i.e., HepG2 and HuH-7 cells. miRNAs were detectable in all samples, but with some variations (Figure 2a). SP-AS was also detectable in all liver cells, although at lower level, as generally observed for many lncRNAs; the highest expression level was observed in HuH-7 cells, where both miRNAs had an intermediate level of expression (Figure 2b). We decided to perform most in vitro experiments on HuH-7 cells, where SP-AS expression and experimentally induced variations should be more easily detectable and quantifiable.

### 2.2. SP-AS Binds to miR-125a and let-7e

**Figure 3:**
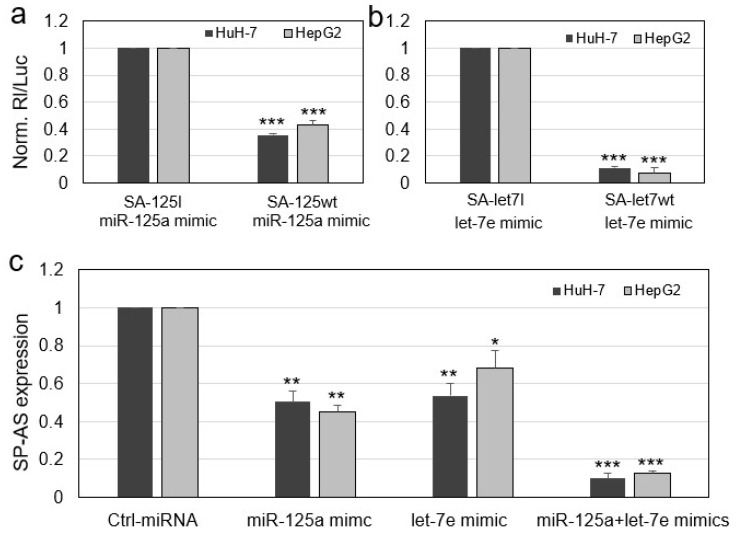
Validation of the binding between SP-AS and miR-125a or let-7e sequences. (**a**,**b**) Renilla luciferase reporter vectors carrying a control inverted target sequence (SA-125I or SA-let-7I) or the SP-AS target sequence for miR-125a or let-7e (SA-125wt or SA-let7wt) were co-transfected in HuH-7 or HepG2 cells along with miR-125a or let-7e mimics. Renilla luciferase activity (Rl) was normalized to that of firefly luciferase activity (Luc), whose coding sequence is carried by the same reporter vector, and the uninhibited activity related to the controls was set to 1. (**c**) Endogenous SP-AS expression was evaluated by RT-QPCR 48 h after transfection of HuH-7 or HepG2 cells with an unrelated miRNA mimic (Ctrl-miRNA) or miR-125a mimic, and let-7e mimic or both miRNA mimics. Data are reported as mean ± s.d. of replicate analyses. * *p* < 0.01, ** *p* < 0.01, *** *p* < 0.001 at Student’s *t*-test by comparison of indicated data sets to their respective controls.

With the aim to experimentally validate the possible binding of either miR-125a or let-7e to SP-AS, the SP-AS complementary sequences to the miRNAs were subjected to a validation test, based on luciferase reporter constructs transfected into the HuH-7 hepatocarcinoma cell line. In detail, those sequences were individually cloned downstream the Renilla reniformis luciferase (Rl) coding sequence carried by psi-Check-2 vector to generate the SA-125wt or SA-let7wt constructs. The reporter plasmids were then transfected into HuH-7 cells along with miRNA mimics and the luciferase activity was measured 48 h after the transfection. The rationale for using the luciferase-based assay is that the potential binding of the miRNA to the SP-AS target, when transcribed together with the luciferase coding sequence, will interfere with the production of the reporter protein, thus reducing the luciferase activity with respect to the controls. The controls are represented by the reporter vectors containing the inverted target sequences (SA-125I or SA-let-7I), that should result uninhibited, thus allowing to register the maximum value of the uninhibited reporter activity. In this experimental setting, both miR-125a and let-7e mimics drastically reduced the activity of the reporter vectors carrying the wild-type target sites (Figure 3a,b), since the registered activity was approximately 48% and 11%, respectively, in comparison to that obtained with their mutant reporter controls, demonstrating a direct interaction between the miRNAs and their target SP-AS sequences. Similar results were obtained by transfecting HepG2 cells (Figure 3a,b).

The described assays are based on the silencing effect on the translation of a chimeric mRNA composed of the Renilla luciferase coding sequence and a segment of SP-AS. In order to assess whether the miRNAs could be able to bind to the natural transcript SP-AS and interfere with its expression, HuH-7 cells were transfected with miRNA mimics, singularly and in combination, and their effect was evaluated on SP-AS expression. QPCR analyses showed that miR-125a mimic and let-7e reduced SP-AS at approximately the same level (50% and 53%, respectively) in comparison to control cells transfected with an unrelated miRNA mimic control; when miR-125a and miR-let7e mimics were co-transfected, SP-AS resulted even more reduced (90% of inhibition) (Figure 3c). It should be noted that miRNA mimics acted as effective siRNAs, since the full sequence complementary pairing and thus knocking-down SP-AS expression very efficiently. Comparable data were obtained by performing experiments into HepG2 cells (Figure 3c).

Overall, these results clearly indicate that miR-125a and let-7e are able to bind their target sequences in the long non-coding RNA SP-AS, whose expression resulted inhibited in hepatocarcinoma cells.

### 2.3. SP-AS Inhibits miR-125a and let-7e Expression and Silencing Activity

**Figure 4:**
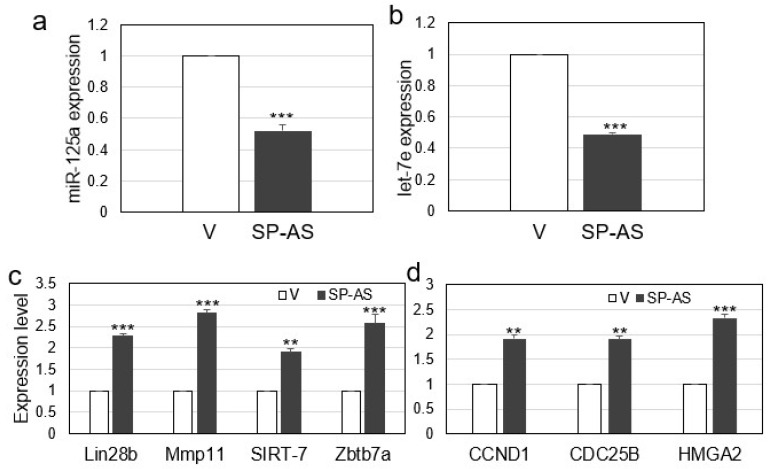
Effect of SP-AS overexpression on miR-125a and let-7e expression and their silencing activity. (**a**) miR-125a and (**b**) let-7e expression was evaluated after transfection of the SP-AS overexpressing vector (SP-AS) or the parental vector (V). (**c**,**d**) In the same transfection points of (**a**) and (**b**), the expression of the main targets of miR-125a and let-7e was evaluated. Data are reported as mean ± s.d. of replicate analyses. ** *p* < 0.01, *** *p* < 0.001 at Student’s *t*-test.

The experiments reported in the above paragraph show that miR-125a and let-7e could bind their complementary sequences within the SP-AS, and the boosting of their expression by miRNA mimic transfection resulted in the inhibition of the long non-coding RNA. We speculated that SP-AS itself, by binding to the miRNAs with perfect complementary target sequences, could reduce their expression and/or their silencing activity on specific target transcripts. With the aim to observe a possible effect of SP-AS on miR-125a and let-7e, a vector overexpressing SP-AS was prepared and transfected into HuH-7 cells. Experimental results showed that miR-125a and let-7e expression were reduced up to approximately 50% as a consequence of SP-AS overexpressing vector transfection in comparison to the control, represented by the transfection of the empty vector (Figure 4a,b).

Given that miRNA levels were reduced by SP-AS ectopic expression, their targets should result upregulated. To test this hypothesis, we decided to evaluate the expression of experimentally validated targets related to cell proliferation and HCC. In particular, for miR-125a, we selected Lin28b, MMP-11, SIRT-7, and Zbtb7a, well established targets with pivotal roles in HCC [25,27,29,30]; for let-7e, we selected Cyclin D1 (CCND1) and D2 (CCND2), validated targets in mouse hepatoblasts, but with conserved targeted sequence in humans, and HMGA2 and CDC25B recently validated in HCC [34,35]. Of note, Lin28 is a target for both miRNAs [29,35]. In the same transfection samples of Figure 4a,b, QPCR analyses revealed that the selected targets resulted upregulated, ranging from 1.9 to 2.6 fold change as a consequence of SP-AS ectopic expression in comparison to the control (Figure 4c,d). CCND2 resulted not detectable under our experimental setting.

Overall, the results indicated that the long non-coding RNA SP-AS binds to the miRNAs, reducing their inhibitory effect on the downstream oncogenic targets, that indeed resulted upregulated.

### 2.4. SP-AS Overexpression Counteracts Antiproliferative Activity of the miRNAs

**Figure 5:**
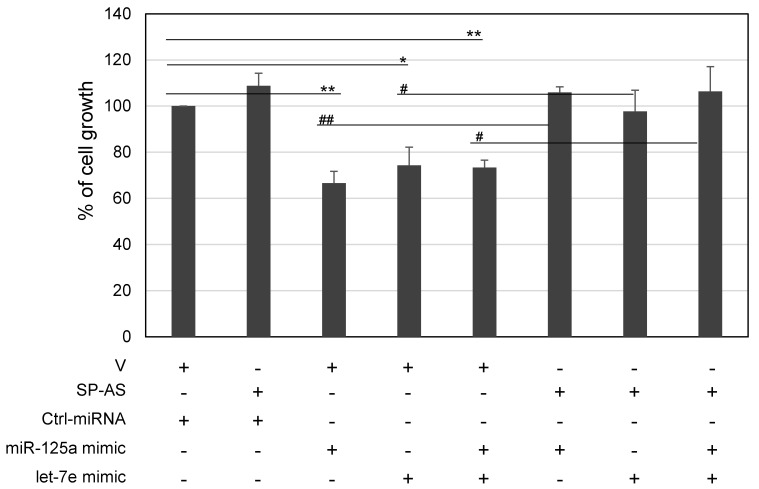
Effect of SP-AS overexpression on the antiproliferative activity of miR-125a and let-7e. HuH-7 cells were transfected with miRNA mimics and their antiproliferative activity was evaluated with or without ectopic expression of SP-AS by MTT assays. Cell growth of cells transfected with control molecules (parental vector, V and unrelated miRNA mimic, Ctrl-miRNA) was set to 100; data are reported as mean ± s.d. of replicate analyses. *p* values at Student’s *t*-test between the indicated experimental groups were * or ^#^ *p* < 0.05; ** or ^##^ *p* < 0.01.

Given that different miRNA targets related to cell proliferation in the HCC context resulted upregulated as a result of SP-AS sponging activity, a number of cell proliferation assays were performed after transfecting SP-AS and miRNA mimics in different combinations. Transfection of miR-125a mimic, or let-7e mimic or both mimics showed a strong antiproliferative effect (ranging from 34% for miR-125a to 26%), consistently with already reported [24]; SP-AS overexpression slightly increased cell growth, although not significantly; however, in combination with miRNA mimics, it was able to revert their antiproliferative effect (Figure 5).

### 2.5. Reverse Expression of SP-AS, miR-125a, and Its mRNA Targets in HCC Samples

Cell culture experiments revealed a possible regulatory network between the long non-coding RNA and the miRNAs, since the expression unbalancing of the one or of the others produces reciprocal effects. Given the oncosuppressive activities of miR-125a and let-7e, the regulatory pathway highlighted by the in vitro studies may have implications for the pathogenesis of HCC. In order to investigate this hypothesis, we inquired the expression of both SP-AS and miRNAs in HCC tissues compared to normal livers by exploiting the expression data contained in The Cancer Genome Atlas (TCGA) in Encori platform, relative to a very large cohort of patients [40]. Comparison of the expression data between 374 HCC samples and 50 normal livers revealed a significant downregulation of miR-125a, with a fold change of 0.71, and a significant reverse expression pattern for SP-AS, that resulted upregulated, with a fold change of 1.57 (Table 1). Unexpectedly, let-7e expression resulted slightly upregulated, although not statistically significant. All miR-125a targets showed a converse expression to the miRNA, since they appeared significantly upregulated in HCC tissues in comparison to normal livers; with regard to let-7e targets, with the exception of CCND1, they were also found upregulated, suggesting the prevailing of other regulatory mechanisms. A reverse correlation between the expression of SP-AS and miR-125a according to the Pearson correlation coefficient was not observed (*r* = 0.498), probably due to the large heterogeneity of tumor stages; however, a positive correlation occurs between SP-AS and all other mRNAs sharing miR-125a binding (Supplementary Table S1).

Overall, the RNA regulatory network among the lncRNA SP-AS, miR-125a, and their mRNA targets, first unveiled in cell culture experiments, could be operative also in HCC and partly contribute to their deregulation with pathological consequences.

## 3. Discussion

High-throughput sequencing technologies and computational platforms have shed light on a plethora of different ncRNA species. LncRNAs constitute the largest class of ncRNAs of mammalian genome, and can be further classified as long intergenic ncRNAs (lincRNAs), antisense RNAs (asRNAs), pseudogenes, and circular RNAs (circRNAs). There is now increasing evidence pointing towards their functional interactions to give rise to complex RNA-based regulatory networks implicated in a wide range of biological and pathological processes, including HCC [19,41]. In particular, ncRNAs can be involved in competitive regulatory interactions, known as competing endogenous RNA (ceRNA) networks, ceRNET, whereby miRNAs and lncRNAs modulate each other, and coding or non-coding transcripts compete for binding to shared miRNAs, thus titrating miRNA availability, and resulting themselves differently modulated [18,19,20]. A disequilibrium in this network, e.g., aberrant expression of any components of the network, could result in a differential modulation of RNA molecules, with pathological consequences.

In this work, we unveiled a novel RNA regulatory network that may have implication for HCC. The main players are an antisense RNA, SP-AS, miR-125a, and let-7e, all transcribed from the same locus (Figure 1), and the miRNA targets. SP-AS is transcribed in the opposite direction of SPACA6 coding transcript, whose function was only recently investigated in vivo in mice [36,37,38]; SP-AS function is still largely unknown and should be addressed in the future, mainly by in vivo study. Intriguingly, it was reported for humans but not mice by a NGS study from Chang et al. (2015) [39]. Here, we were able to detect SP-AS expression by QPCR in HuH-7 cells, but also in HepG2 cells and human primary hepatocytes (Figure 2) and isolate the transcript. MiR-125a, a homologue of the first discovered miRNA lin-4, and let-7e are well known miRNAs, with pivotal function in development and cell differentiation, but also in cancer, including HCC [42]. The sequence complementary between SP-AS and the miRNAs prompted us to investigate their possible reciprocal interactions. First, we experimentally validated their interaction by a luciferase assay; then, we observed that the boosting of miRNAs by miRNA mimic transfection resulted in SP-AS reduced expression, up to 10% of residual expression in comparison to the control when both miRNA mimics were used (Figure 3). The inhibition of expression was reciprocal, since the overexpression of SP-AS reduced miR-125a and let-7e at comparable level of approximately 50% (Figure 4a,b). Then, we inquired about the effects of overexpression of the SP-AS on the miRNA targets that were experimentally validated in the HCC context and related to cell proliferation. The common target Lin28b resulted upregulated, as well as all the other mRNAs (Figure 4c,d). Cell proliferation assays confirmed that SP-AS was able to counteract antiproliferative activity of the miRNAs (Figure 5).

Several recent studies have started to explore ceRNA interactions by exploiting the large datasets from The Cancer Genome Atlas (TCGA) database [40,43]. We used a similar approach to investigate possible differential expression profiling between 374 HCC tissues and 50 normal livers. As reported in Table 1, SP-AS and miR-125a showed reverse expression; in particular, miR-125a is downregulated of approximately 30%, consistent with our studies on a smaller patient cohort and all its targets resulted upregulated, indicating that this ceRNET could be operative and partly participate in hepatocarcinogenesis. Surprisingly, let-7e expression did not significantly change between cancer and normal tissues, whereas some of its targets resulted upregulated. These discrepancies with data collected in vitro could depend on the occurrence of other prevailing regulatory mechanisms during the hepatocarcinogenesis; an alternative explanation may be found in the samples heterogeneity of HCC stages that allow us to analyze the “mean” level of a specific transcript in the entire pool, not correlated to a specific tumor stage. This last consideration may also explain the observed weak Pearson correlation coefficients.

**Figure 6:**
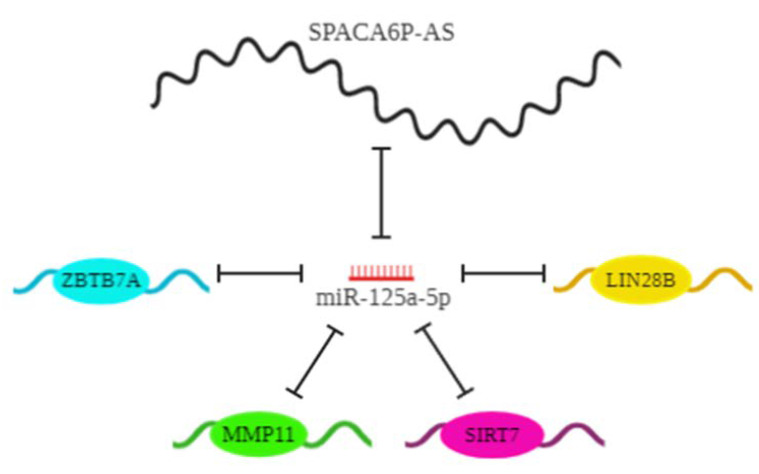
Interactions among SP-AS, miR-125a, and downstream target mRNAs depict a competing endogenous RNA network (ceRNET) in HCC cells. The long non-coding RNA (lncRNA) SP-AS and miR-125a regulate each other; SP-AS can interact with the miRNA, preventing its inhibitory binding to the target transcripts encoding proteins, represented by ovals; the mRNA targets can compete with one other for binding to the miRNA and titrating its availability, thus having a regulatory potential, beyond their coding power. Finally, both Zbtb7a and Lin28b proteins can transcriptionally or post-transcriptionally repress miR-125a, giving rise to a multifaceted liaison between biogenesis and regulatory role of the miRNA. Aberrant expression of any component of the circuit could derail the network, with pathological consequences.

Intriguingly, some of miR-125a targets add another layer of complexity to the network constituted by SP-AS/miR-125a/mRNA targets (Figure 6); in fact, Zbtb7a and Lin28b regulate miR-125a at transcriptional and post-transcriptional level, respectively. Zbtb7a (also known as Pokemon) is a transcriptional factor with an oncogenic role in HCC and it is able to repress the transcription of miR-125a by binding to a specific sequence in its promoter [30,31,44,45]. Lin28 is considered a gatekeeper molecule that regulates the transition between pluripotency and commitment to differentiation during the development; mirroring the development, many cancer types showed a reactivation of Lin28, including HCC [42,46]. In particular, in mice the overexpression of Lin28b, the main liver isoform, is able to initiate hepatocellular carcinoma [47]. Lin28b represses miR-125a biogenesis by binding to a specific sequence of the pre-miRNA [29]. Overall, both Zbtb7a and Lin28b, part of the ceRNET unveiled in this work, could themselves contribute to the downregulation of miR-125a expression observed in HCC tissues, and together constitute a piece of a puzzle, where different mechanisms are needed to complete the picture and whose unbalancing could be a driving force for HCC.

Finally, it has not escaped our attention that also miR-99b could have a role in balancing the interactions depicted in Figure 6, thus deserving future investigations. In fact, miR-99b sequence is fully complementary to the 5′ splice junction of the intron of SP-AS (Figure 1), spanning positions-6 to + 16 with respect to the splice junction, possibly forming a 22 nt duplex. On the basis of its genomic location, a competition between SP-AS splicing and miR-99b biogenesis could be envisaged [48,49]; importantly, it has been hypothesized that miR-99b competes with the binding of U1 and U6 snRNPs required for SP-AS splicing [50,51]. Supporting this hypothesis, Mahla-Aviv et al. found miR-99b sequences into HeLa cell supraspliceosomes, nuclear dynamic machines in which a crosstalk between pre-mRNA splicing and nuclear maturation of miRNAs probably occurs [50].

A deeper insight into all those RNA regulatory networks could unravel previously unappreciated layers of complexity and regulation, with an impact on the physiological process, such as development and cell differentiation, taking into account the physiological role of miR-125a and let-7e, but also on diseases pathogenesis, such as cancer, taking into account the tumor suppressor role of the miRNAs. Furthermore, exploration of RNA interactions could be exploited in the future to identify new biomarkers and therapeutic targets, as well as for development of innovative therapeutic tools, e.g., for restoring the expression of tumor suppressor miRNAs by unbalancing the identified ceRNET.

## 4. Materials and Methods

### 4.1. DNA Constructs

Luciferase reporter constructs were prepared as follows: the SP-AS sequences complementary to miR-125a or let-7e were obtained by chemical synthesis of complementary oligonucleotides (Invitrogen) containing upstream *Xho*I and *Eco*RV restriction sites and a downstream *Not*I site and cloning in psiCheck-2 (Promega) [52]. In brief, each couple of oligonucleotides, representing the SP-AS target sites for the two miRNAs, was annealed, 5′-phosphorylated with T4 polynucleotide kinase (Promega), and ligated into *Xho*I and *Not*I sites of psiCheck-2. Digestion with *Eco*RV were then used to screen the recombinant clones, denominated SP-125wt or SP-let7wt. Control plasmids were obtained by the same approach, i.e., cloning the couples of oligonucleotides representing the inverted target sequence for either miR-125a (SP-125I) or let-7e (SP-let7I) in psiCheck-2 vector.

SP-AS overexpressing vector was prepared by RT-PCR amplification of SP-AS followed by the cloning of the PCR product in pcDNA3.1 (Invitrogen). In detail, a PCR was carried out on cDNA from HuH-7 cells with a forward primer (AS-F: 5′-CCCAAGCTTGGGTCAGAGGTCAGGTTTC-3′) containing the underlined *Hind*III restriction site, and a reverse primer (AS-R: 5′-CGGAATTCCTGGTCCCTGTCTGTCTG-3′) containing the underlined *Eco*RI restriction site; then, PCR product was digested and cloned into *Hind*III/*Eco*RI restriction sites of pcDNA3.1.

All the constructs were sequenced to confirm their identity.

### 4.2. Cell Culture, Transfections, and Luciferase Assay

Human liver cancer cell lines HepG2 and HuH-7 were cultured in RPMI 1640 and DMEM, respectively, containing 10% fetal bovine serum, 2 mM l-glutamine, 50 U/mL penicillin, and 100 μg/mL streptomycin. The day before transfection, cells were trypsinized and seeded in medium without antibiotics in 12-well plates. Transfections were then performed with cells at 80–90% of confluence by using 3 μL of Lipofectamine2000 (Invitrogen, Waltham, MA, USA) for 1 μg of nucleic acids, as described by the manufacturer. In particular, HuH-7 cells were transfected with 0.05 μg of reporter constructs (SP-miR125wt, SP-miR125I, SP-let7wt, SP-let7I); miR-125a mimic, let-7e mimic, or their control (Ctrl-miRNA) with unrelated sequence (all from Dharmacon, Lafayette, CO, USA) were transfected or co-transfected at 50 nM; 1.5 μg of SP-AS plasmid or the parental vector pcDNA3.1 were used for SP-AS overexpression experiments performed on 12-well plates, and 200 ng of both plasmids were used for MTT assays performed on 96-well plates. After 6 h, transfection mix was replaced with complete medium. The analyses were performed 48 h after transfection.

Luciferase activity was recorded using the Dual-Luciferase Reporter Assay System (Promega, Madison, WI, USA), according to the manufacturer’s protocol.

### 4.3. RNA Purification and Real-Time PCR Analyses

Total RNA was extracted from cell cultures and from hNHEPS^®^ Human Hepatocytes (Lonza, Basel, Switzerland) by miRNeasy mini kit (Qiagen, Hiden, Germany). MicroRNA-125a and Let-7e were quantified along with RNU6B (reference transcript) by RT-qPCR with TaqMan^®^ miRNA assays (Cat. n. 4427975; miR-125a-5p, ID: 002198; let-7e-5p, ID: 002406; RNU6B, ID: 001093) from Applied Biosystems according to the manufacturer’s protocol.

For quantification of the other transcripts, total RNA was retrotranscribed by SensiFAST™ cDNA Synthesis kit (Bioline). Then standard SYBR Green Real-time qPCR assays were performed with the following primers: SP-AS, 5′-CAAATGTCATGCTCTGGAGGA-3′ and 5′-CCTGAGACCCTTTAACCTGTG-3′; Lin28b, 5′-AGAAAAGAAAACCAAAGGGAGATAG-3′ and 5′-GAGGTAGACATACTTCCTTAGCATGA-3′; MMP11, 5′-TCCTGACTTCTTT GGCTGTG-3′ and 5′-CCATGGGTCTCTAGCCTGAT-3′ [23]; SIRT-7, 5′-GTCTGCATGAGCAGAAGCTG-3′ and 5′-GGAACGCAGGA GGTACAGAC-3′; Zbtb7a, 5′-ACGAGTGCAACATCTGCAAG-3′ and 5′-GGTCGTAGTTGTGGGCAAAG-3′ [30]; CDC25B, 5′-TCAAGATGCCATGGAAGCCC-3′ and 5′-TCCATCTTCCGGTCAGGACT-3′ [34]; HMGA2, 5′- AAGCAGCAGCAAGAACCAAC-3′ and 5′- GCCTCTTGGCCGTTTTTCTC-3′ [34]; CCND1, 5′-TGGCGTTTCCCAGAG TCATC-3′ and 5′-AAGGAAGGGGCAGGGGATAA-3′; GAPDH (reference transcript), 5′-GAAGGTGAAGGTCGGAGTC-3′ and 5′-GAAGATGGTGATGGGATTT-3′.

The expression levels of miRNAs and transcripts were normalized to their respective reference genes by using the 2-^ΔCt^ method.

### 4.4. Cell Proliferation Assays

HuH-7 cells were plated in 96-well microtiter plates, transfected as detailed above and 48 h later their growth was evaluated by quadruplicate analysis with MTT assay. In brief, 50 μL of 1 mg/mL 3-(4,5-Dimethylthiazol-2-yl)-2,5-Diphenyltetrazolium bromide (MTT) were mixed with 200 μL of medium and added to the well. After 1 h of incubation at 37 °C, the medium was removed, and the purple formazan crystals produced in the viable cells were solubilized in 100 μL of dimethyl sulfoxide and quantitated by measurement of absorbance at 570 nm with a plate reader.

### 4.5. Statistical and Bioinformatic Analyses

Comparison of data sets in the different experiments was performed by Student’s *t*-test and value of *p* < 0.05 was considered statistically significant.

Expression data from HCC patients were analyzed on ENCORI platform (The Encyclopedia of RNA Interactomes, http://starbase.sysu.edu.cn) based on the expression values of genes and miRNAs from RNA-seq and miRNA-seq data of The Cancer Genome Atlas (TCGA) [40], collecting samples from different ethnicity and different tumor stages, ranging from Stage I (approximately the half) to IV. In particular, in the section Pan-Cancer of Encori platform, we obtained the fold change expression and *p* values between HCC tissues compared to normal livers from the two subsections “miRNA differential expression” and “Gene differential expression”, for miRNA molecules and for SP-AS and target mRNAs, respectively. Pearson correlation coefficients were obtained from “miRNA-Target CoExpression” (miR-125a and SP-AS) and “RNA-RNA CoExpression” (SP-AS and the other miR-125a targets) subsections, where expression values of genes were scaled with log2 (FPKM + 0.01) and miRNAs scaled with log2 (RPM + 0.01).

## Figures and Tables

**Table 1:** Gene expression changes in 374 HCC tissues compared to 50 normal liver samples (data from Encori, TCGA).

	Fold Change	*p*-Value
SPACA6P-AS	1.57	0.0013
miR-125a-5p	0.71	1.8 × 10^−7^
let-7e-5p	1.22	0.97
Lin28b	55.81	1.1 × 10^−5^
MMP11	22.28	2.3 × 10^−30^
SIRT7	3.15	1.1 × 10^−35^
Zbtb7a	1.43	3.1 × 10^−8^
CCND1	1.12	0.094
CDC25B	3.26	1.3 × 10^−17^
HMGA2	21.88	0.0006

## Supplementary Materials

The following are available online at https://www.mdpi.com/1422-0067/21/14/5068/s1, Table S1: Pearson correlation coefficients (r) between indicated molecules in HCC tissues (data from Encori, TCGA).
