# Supplementary material for: A Novel ceRNA Regulatory Network Involving the Long Non-Coding Antisense RNA SPACA6P-AS, miR-125a and its mRNA Targets in Hepatocarcinoma Cells
Source: Int J Mol Sci. 2020 Jul 17;21(14):5068. doi: 10.3390/ijms21145068 (PMC7404396; doi:10.3390/ijms21145068)
Supplement: Supplementary file 1 [file ijms-21-05068-s001.pdf]

Table S1. Pearson correlation coefficients (r) between indicated molecules in HCC tissues (data from Encori, TCGA).

	r	***p*-Value**
SP-AS/125a	0.498	1.56×10^{-24}
SP-AS/Lin28b	0.174	7.36×10^{-4}
SP-AS/MMP11	0.358	9.36×10^{-13}
SP-AS/SIRT7	0.240	2.72×10^{-6}
SP-AS/Zbtb7a	0.204	7.29×10^{-5}
